# Long-Term Ozanimod Therapy in Patients With Moderately Active Ulcerative Colitis After Failure of 5-Aminosalicylic Acid

**DOI:** 10.1093/ibd/izaf195

**Published:** 2025-09-26

**Authors:** Andres Yarur, Peter Irving, Britta Siegmund, Marla C Dubinsky, Ashwin N Ananthakrishnan, Miguel Regueiro, Ryan C Ungaro, Timothy Ritter, Hiroshi Nakase, Zhaohui Liu, Dimpy Mehra, Mark T Osterman, Anjali Jain, David T Rubin, Toshifumi Hibi

**Affiliations:** Cedars-Sinai Medical Center, Los Angeles, CA 90048, United States; Guy’s and St Thomas’ NHS Foundation Trust, London SE1 7EH, United Kingdom; Department of Gastroenterology, Infectious Diseases and Rheumatology, Charité – Universitätsmedizin Berlin, corporate member of Freie Universität Berlin, Humboldt-Universität zu Berlin, 10117 Berlin, Germany; Susan and Leonard Feinstein Inflammatory Bowel Disease Clinical Center, Icahn School of Medicine at Mount Sinai, New York, NY 10029, United States; Massachusetts General Hospital, Boston, MA 02114, United States; Cleveland Clinic, Cleveland, OH 44195, United States; Icahn School of Medicine at Mount Sinai, New York, NY 10029, United States; GI Alliance, Southlake, TX 76092, United States; Sapporo Medical University, Sapporo, Hokkaido 060-8556, Japan; Bristol Myers Squibb, Lawrence Township, NJ 08648, United States; Bristol Myers Squibb, Lawrence Township, NJ 08648, United States; Bristol Myers Squibb, Lawrence Township, NJ 08648, United States; Bristol Myers Squibb, Lawrence Township, NJ 08648, United States; Inflammatory Bowel Disease Center, University of Chicago Medicine, Chicago, IL 60637, United States; Center for Advanced IBD Research and Treatment, Kitasato Institute Hospital, Kitasato University, Tokyo 108-8642, Japan

**Keywords:** ozanimod, advanced therapy–naive, ulcerative colitis

## Abstract

**Background:**

We evaluated the efficacy and safety of ozanimod after 5-aminosalicylic acid (5-ASA) failure in advanced therapy (AT)–naive patients with moderate ulcerative colitis (UC) in True North and its open-label extension (OLE).

**Methods:**

True North was a randomized, 52-week, phase 3 trial with an optional OLE. Efficacy was assessed in True North and the OLE; safety was assessed through OLE week 190.

**Results:**

Overall, 203 AT-naive True North patients had moderate UC (Mayo endoscopic subscore of 2 + modified Mayo score of 4-6 + rectal bleeding subscore ≥1). Of these, 139 were also immunomodulator-naive and not receiving corticosteroids (5-ASA–exposed only) at baseline. Patients with moderate UC receiving ozanimod vs placebo achieved greater efficacy rates for all week 10 and week 52 outcomes, regardless of prior immunomodulator/corticosteroid use (eg, week 10 clinical remission: AT-naive = 36.8% vs 10.6%; 5-ASA–exposed only = 37.9% vs 17.2%). Higher symptomatic response rates were achieved by week 2 with ozanimod in AT-naive patients with moderate UC vs the overall AT-naive population (50.5% vs 38.7%); similar trends were observed in patients exposed only to 5-ASA. Efficacy was maintained through OLE week 190 in patients who entered OLE as True North week 52 ozanimod clinical responders. Of those entering OLE as True North week 10 ozanimod clinical nonresponders, 69.0% of AT-naive patients and 68.4% of patients exposed only to 5-ASA achieved symptomatic response by week 5. No new safety signals emerged.

**Conclusions:**

Ozanimod was safe, effective, and durable up to ∼5 years in AT-naive patients with moderate UC who failed conventional therapy. ClinicalTrials.gov: NCT02435992, NCT02531126.

Key Messages **What is already known?**Effective and safe alternative therapies are needed in patients with mild/moderate ulcerative colitis (UC) who experience disease progression despite first-line 5-aminosalicylic acid (5-ASA) treatment.

**What is new here?**Ozanimod was safe and effective for up to ∼5 years in advanced therapy–naive patients with moderate UC who failed conventional therapy, including those who started ozanimod immediately after 5-ASA failure without initiating immunomodulators or corticosteroids.

**How can this study help patient care?**Early initiation of ozanimod may be beneficial as a first-line advanced therapy in patients with moderate UC in whom 5-ASA fails.

## Introduction

Ulcerative colitis (UC) is a chronic immune-mediated inflammatory condition of the gastrointestinal tract.^1–3^ Although 5-aminosalicylic acids (5-ASAs) are used as the first-line treatment option in patients with mild to moderate UC,^1^^,^^4^ a subset of these patients experience progression to moderate ­to severe UC despite 5-ASA treatment.^4^ Additionally, patients may be intolerant of 5-ASA or have difficulty adhering to long-term treatment.^5^^,^^6^ As a result, these patients often receive conventional treatment escalation with add-on corticosteroids or thiopurines.^4^^,^^7–9^ However, long-term corticosteroid use is not recommended by current guidelines because of the risk of serious complications (ie, infection, bone, and metabolic adverse events [AEs]),^1^^,^^10^ and thiopurines lack induction efficacy and are associated with significant side effects.^7^ There is a need for effective and well-tolerated alternatives to steroids and thiopurines in patients who failed 5-ASA therapy.

The sequencing of approved advanced therapies (ATs) in UC is evolving.^7^^,^^9^^,^^11^ The large number of ATs available contributes to the difficulty in determining appropriate therapy sequencing for maximum benefit.^11^^,^^12^ In addition, patients with prior exposure to ATs may be less likely to respond to subsequent ATs, and it is not known whether or not a medication’s mechanism of action should influence sequencing.^11^^,^^13–17^

Minimal data are available on early AT intervention in patients with moderate UC, partly because moderate UC is not classified as its own category, and most clinical trials included broad and heterogeneous patient populations with either mild to moderate or moderate to severe disease.^1^^,^^18^ More AT data, specifically in a population of patients with moderate UC, are needed, as disease severity and disease activity may impact treatment response as well as the willingness of patients to initiate an AT. Moderate UC is defined by various criteria, including endoscopic disease activity with or without patient-reported outcomes.^1^^,^^19^ Orally administrated effective ATs with acceptable safety profiles are needed as an alternative to currently available treatments.

Ozanimod, a selective sphingosine 1-phosphate (S1P) receptor 1 and 5 modulator, is an oral small molecule approved for the treatment of moderate to severe UC.^20–22^ Ozanimod was efficacious and well tolerated for up to 52 weeks in patients with moderate to severe UC in the phase 3 True North study (NCT02435992).^23^ Previous analyses of True North and the True North open-label extension (OLE) (NCT02531126) demonstrated that ozanimod was efficacious, durable, and safe for up to 2 years in AT-naive patients.^24^ This post hoc analysis of the phase 3 True North and OLE studies further expands on previous studies but focuses on the efficacy and safety of ozanimod in the AT-naive patients with UC who had moderate UC immediately after nonresponse to 5-ASA only or after nonresponse to 5-ASA and immunomodulators and/or concomitant corticosteroids.

## Methods

### Study design

The True North^23^ and True North OLE^25^ study designs have been previously described (Supplemental Figure 1, Supplemental Methods). True North was a randomized, double-blind, placebo-controlled, 52-week phase 3 study that evaluated the efficacy and safety of ozanimod 0.92 mg in patients with moderate to severe UC.^23^ In the 10-week induction period, patients in cohort 1 were randomized in a double-blind manner to receive once-daily ozanimod or placebo and patients in cohort 2 received once-daily open-label ozanimod.^23^ Patients receiving ozanimod in either cohort 1 or 2 with clinical response at week 10 were rerandomized to receive either ozanimod or placebo in the subsequent maintenance period.^23^ Eligible patients were able to enroll in an optional OLE.^23^^,^^25^

### Patients

This analysis included efficacy and safety data from patients with moderate UC who were AT-naive (ie, naive to biologics and Janus kinase inhibitors). A subanalysis of this patient population focused on patients who were also naive to immunomodulators without concomitant corticosteroid use at baseline (ie, those who were on 5-ASA and had active moderate disease while on 5-ASA). Moderate disease was defined as Mayo endoscopic subscore (MES) of 2 combined with the modified Mayo score (MMS; sum of the rectal bleeding subscore [RBS], stool frequency subscore [SFS], and MES scores) of 4 to 6 and RBS ≥1. Sensitivity analyses were conducted using 2 additional definitions of moderate disease in AT-naive patients: an MES of 2 alone or an MES of 2 combined with a total Mayo score (sum of the RBS, SFS, MES, and Physician Global Assessment subscore) of 6 to 9 and RBS ≥1. Efficacy outcomes were also assessed in the overall AT-naive population of patients with moderate to severe UC and in the overall population of patients with moderate to severe UC exposed only to 5-ASA.

### Efficacy outcomes

RBS and SFS were self-reported and collected in an electronic diary throughout the study. Endoscopy and biopsy samples were centrally assessed by blinded central readers.^23^ All efficacy outcomes assessed in this analysis are defined in Supplemental Table 1.

Symptomatic response and remission were evaluated from weeks 2 to 10 in the True North induction period to assess onset of action. Clinical remission, clinical response, endoscopic improvement, histologic remission, and mucosal healing were assessed at True North week 10; these endpoints, along with corticosteroid-free remission, were also assessed at True North week 52.

Long-term efficacy was assessed over approximately 5 years of ozanimod treatment in the subset of patients with clinical response at week 52 who received continuous ozanimod throughout True North and who subsequently entered the OLE. Clinical remission, clinical response, endoscopic improvement, corticosteroid-free remission, histologic remission, mucosal healing, and endoscopic remission were assessed through OLE week 190 (ie, ∼4 years of ozanimod exposure in the OLE).

Extended induction and delayed response to ozanimod were evaluated in True North week 10 ozanimod clinical nonresponders who were not rerandomized to treatment in the True North maintenance period and instead entered the OLE. Symptomatic response and remission were evaluated at OLE weeks 5 and 10 to assess for efficacy of ozanimod with extended induction.

#### Safety

Safety was assessed throughout the study as in the overall True North population.^23^

### Statistical analysis

In this post hoc analysis of True North and the True North OLE, OLE data were derived from an interim analysis as of January 10, 2024. All patients who received ≥1 dose of the study drug were assessed. Details regarding the statistical analysis of True North have been previously reported and included in the Supplemental Methods.^23^

OLE data were summarized descriptively using observed case (OC) and nonresponder imputation (NRI) analyses, and no hypothesis testing was performed. Safety outcomes were summarized descriptively.^23^

### Ethics

This study adhered to the Good Clinical Practice guidelines and the ethical principles outlined in the Declaration of ­Helsinki. Investigators at each study site obtained protocol and informed consent approval by an institutional review board or independent ethics committee prior to initiating the study. Written consent was obtained from each patient in the study, which was sponsored by Bristol Myers Squibb.

## Results

### Study population

#### Disposition

**Figure 1.**
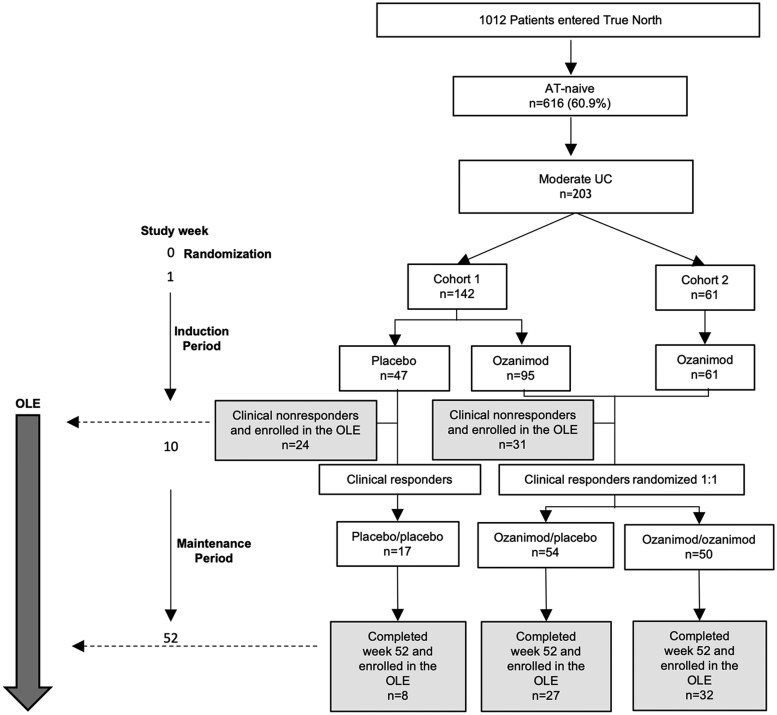
Disposition for the True North advanced therapy (AT)–naive patients with moderate ulcerative colitis (UC). Moderate UC is defined as a Mayo endoscopic subscore of 2 + modified Mayo score of 4-6 + rectal bleeding subscore ≥1. OLE, open-label extension.

In the True North study, 60.9% (n = 616 of 1012) of patients were AT-naive, of whom 33.0% (n = 203 of 616) had moderate UC at baseline using the definition of an MES of 2 combined with an MMS of 4 to 6 and RBS ≥1 (Figure 1).^23^ Most AT-naive patients with moderate UC (68.4% [n = 139 of 203]) were exposed only to 5-ASA at the True North baseline.

Baseline characteristics were similar in patients with moderate UC who were AT-naive and in the subset of patients who were exposed only to 5-ASA (Table 1). These characteristics were similar to those in the overall AT-naive population (regardless of disease severity) and in patients with moderate UC using 2 alternative definitions of moderate disease (Supplemental Tables 2 and 3).

**Table 1.** Baseline demographic and clinical characteristics in patients with moderate UC (MES of 2 + MMS of 4-6 + RBS ≥1).

Characteristics	AT-naive patients with moderate UC (n = 203)			Patients exposed only to 5-ASA (n = 139)		
	Cohort 1		Cohort 2	Cohort 1		Cohort 2
	Placebo (n = 47)	Ozanimod (n = 95)	Ozanimod (n = 61)	Placebo (n = 29)	Ozanimod (n = 66)	Ozanimod (n = 44)
Age, y	41.6 ± 14.6	42.8 ± 13.6	44.2 ± 15.7	42.6 ± 14.3	43.1 ± 13.5	42.9 ± 15.2
Male	27 (57.4)	55 (57.9)	36 (59.0)	14 (48.3)	36 (54.5)	24 (54.5)
Years since UC diagnosis	6.3 ± 7.6	5.8 ± 6.1	6.4 ± 7.6	7.0 ± 9.0	4.8 ± 5.5	6.5 ± 8.0
Extent of UC disease						
Left sided	33 (70.2)	65 (68.4)	46 (75.4)	22 (75.9)	46 (69.7)	35 (79.5)
Extensive	14 (29.8)	30 (31.6)	15 (24.6)	7 (24.1)	20 (30.3)	9 (20.5)
TMS^a^	7.4 ± 0.8	7.3 ± 0.8	7.2 ± 0.8	7.3 ± 0.8	7.3 ± 0.8	7.2 ± 0.8
MMS^b^	5.3 ± 0.7	5.4 ± 0.7	5.1 ± 0.8	5.2 ± 0.8	5.3 ± 0.7	5.2 ± 0.8
MES						
Moderate (MES = 2)	47 (100.0)	95 (100.0)	61 (100.0)	29 (100.0)	66 (100.0)	44 (100.0)
Severe (MES = 3)	0	0	0	0	0	0
Concomitant 5-ASA use	43 (91.5)	91 (95.8)	61 (100.0)	29 (100.0)	65 (98.5)	44 (100.0)
Concomitant CS use at baseline	11 (23.4)	16 (16.8)	11 (18.0)	0	0	0
Prior 5-ASA use	45 (95.7)	93 (97.9)	61 (100.0)	28 (96.6)	64 (97.0)	44 (100.0)
Prior CS use	32 (68.1)	59 (62.1)	36 (59.0)	14 (48.3)	34 (51.5)	19 (43.2)
Prior IMM use	12 (25.5)	16 (16.8)	8 (13.1)	0	0	0

Values are n (%) or mean ± SD.

a Sum of RBS, SFS, MES, and Physician Global Assessment subscore.

b Sum of RBS, SFS, and MES.

Abbreviations: 5-ASA, 5-aminosalicylic acid; AT, advanced therapy; CS, corticosteroid; IMM, immunomodulator; MES, Mayo endoscopic subscore; MMS, modified Mayo score; RBS, rectal bleeding subscore; SFS, stool frequency subscore; TMS, total Mayo score; UC, ulcerative colitis.

### Efficacy

#### Onset of action

**Figure 2.**
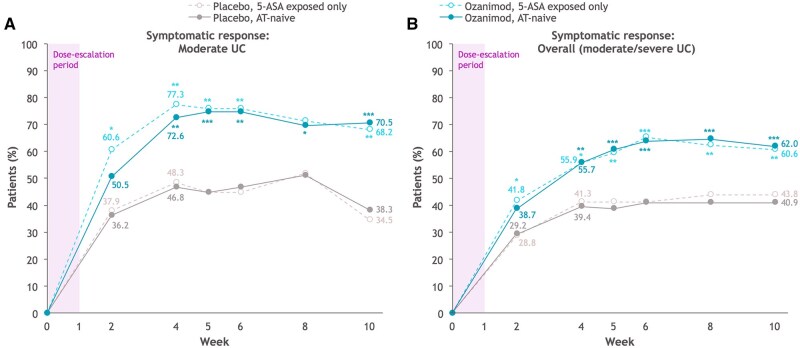
Symptomatic efficacy through week 10 in cohort 1 of the True North induction period in advanced therapy (AT)–naive patients and patients exposed only to 5-aminosalicylic acid (5-ASA). (A) Symptomatic response in AT-naive patients with moderate ulcerative colitis (UC) (Mayo endoscopic subscore of 2 + modified Mayo score of 4-6 + rectal bleeding subscore ≥1). (B) Symptomatic response in the overall AT-naive patients. Shading indicates the 1-week dose escalation period of ozanimod. *P < .05, **P < .01, ***P < .001.

A greater proportion of AT-naive patients with moderate UC achieved symptomatic response as early as 2 weeks after treatment initiation with ozanimod (1 week after dose titration) compared with the overall AT-naive patient population (50.5% vs 38.7%), and results were similar in patients exposed only to 5-ASA (60.6% vs 41.8%) (Figure 2). As reported previously,^24^ all groups showed symptomatic response with ozanimod treatment by week 2, although significant differences between ozanimod and placebo were observed at week 2 in the populations of patients exposed only to 5-ASA (*P *< .05). Similar patterns were seen when alternative definitions of moderate disease were used (Supplemental Figure 2).

#### True North induction efficacy

**Figure 3.**
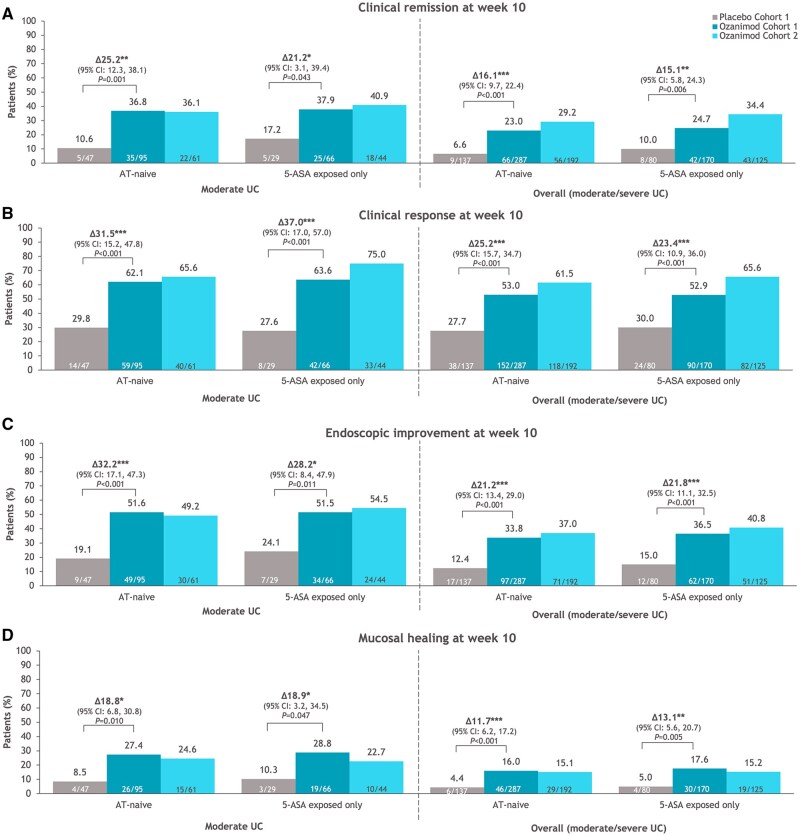
Efficacy outcomes at week 10 in patients with moderate ulcerative colitis (UC; Mayo endoscopic subscore of 2 + modified Mayo score of 4-6 + rectal bleeding subscore ≥1) and in the overall population who were advanced therapy (AT)–naive or exposed only to 5-aminosalicylic acid (5-ASA). (A) Clinical remission. (B) Clinical response. (C) Endoscopic improvement. (D) Mucosal healing. Δ refers to the difference between ozanimod (cohort 1) and placebo in the induction period. *P < .05, **P < .01, ***P < .001 vs placebo. CI, confidence interval.

Patients receiving ozanimod achieved greater efficacy rates vs placebo for all evaluated efficacy endpoints at week 10 across all groups, regardless of baseline disease severity and prior immunomodulator or corticosteroid use (Figure 3; Supplemental Figure 3). In patients with moderate UC receiving ozanimod or placebo, 27.4% and 8.5% of those who were AT-naive and 28.8% and 10.3% of those exposed only to 5-ASA, respectively, achieved mucosal healing at week 10 (Figure 3D). Importantly, absolute proportions and differences in proportions between treatment groups were higher in AT-naive patients with moderate UC compared with the overall AT-naive patient population (Figure 3; Supplemental Figure 3). Efficacy rates at week 10 for all endpoints were similar across all definitions of moderate UC (Figure 3; Supplemental Figure 3). Absolute proportions of patients exposed only to 5-ASA achieving the various clinical and mucosal endpoints at the end of induction were similar or slightly higher than those of the AT-naive patients, regardless of baseline disease severity (Figure 3; Supplemental Figure 3).

#### True North maintenance efficacy

**Figure 4.**
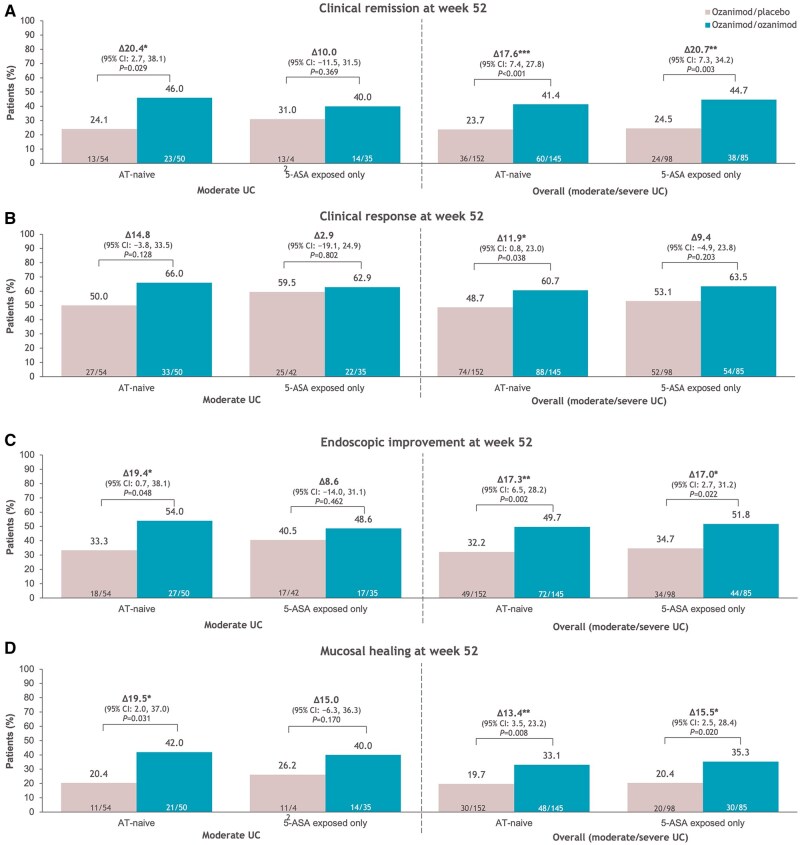
Efficacy outcomes at week 52 in patients with moderate ulcerative colitis (UC; Mayo endoscopic subscore of 2 + modified Mayo score of 4-6 + rectal bleeding subscore ≥1) and in the overall population who were advanced therapy (AT)–naive or exposed only to 5-aminosalicylic acid (5-ASA). (A) Clinical remission. (B) Clinical response. (C) Endoscopic improvement. (D) Mucosal healing. *P < .05, **P < .01, ***P < .001 vs placebo. CI, confidence interval.

Patients with moderate UC who were rerandomized to continue ozanimod achieved greater efficacy rates than those rerandomized to placebo for all evaluated endpoints at week 52 during the maintenance period in both AT-naive groups (Figure 4; ­Supplemental Figure 4). Notably, in patients with moderate UC rerandomized to ozanimod or placebo, 42.0% and 20.4% of those who were AT-naive and 40.0% and 26.2% of those exposed only to 5-ASA, respectively, achieved mucosal healing at week 52 (Figure 4D). Generally similar ozanimod efficacy rates at week 52 were observed in AT-naive patients with moderate UC and in the overall AT-naive patient population; similar trends were observed in patients exposed only to 5-ASA (Figure 4; Supplemental Figure 4). Efficacy was comparable in AT-naive patients and patients exposed only to 5-ASA, regardless of baseline disease severity. Efficacy rates at week 52 for all endpoints were similar across all definitions of moderate UC (Figure 4; Supplemental Figure 4).

#### OLE efficacy

**Figure 5.**
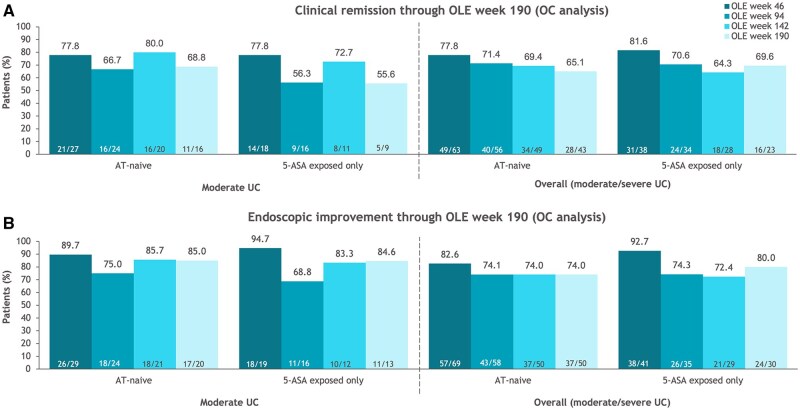
Efficacy through True North open-label extension (OLE) study week 190 in patients with moderate ulcerative colitis (UC; Mayo endoscopic subscore of 2 + modified Mayo score of 4-6 + rectal bleeding subscore ≥1) and in the overall population who were advanced therapy (AT)–naive or exposed only to 5-aminosalicylic acid (5-ASA) and who entered the OLE as True North week 52 ozanimod clinical responders (observed case [OC] analysis). (A) Clinical remission. (B) Endoscopic improvement.

Ozanimod efficacy was maintained through OLE week 190 in AT-naive patients with moderate UC who received 52 weeks of ozanimod treatment in True North and entered the OLE in clinical response (Figure 5; Supplemental Figure 5). Efficacy rates were comparable through OLE week 190 in the moderate and overall groups and in AT-naive patients and patients exposed only to 5-ASA (OC analysis). Results of sensitivity analyses were generally similar using alternative definitions of moderate UC (OC analysis) (Supplemental Figure 5). Similar trends were observed in the NRI analysis (Supplemental Figure 6).

#### Extended induction

**Figure 6.**
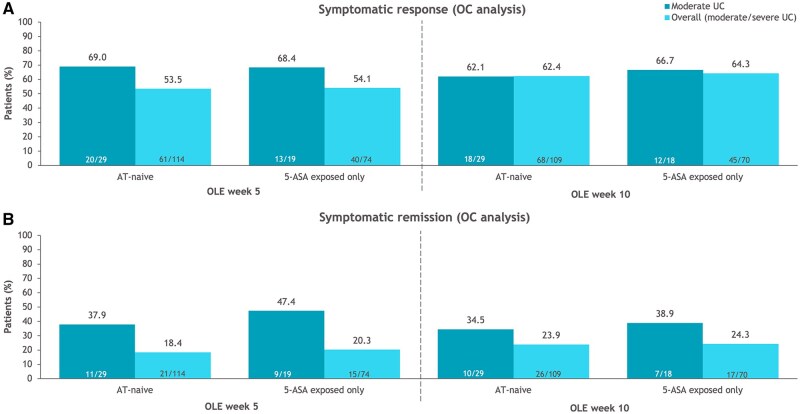
Symptomatic efficacy with up to 10 weeks of extended induction in the True North open-label extension (OLE) study in patients with moderate ulcerative colitis (UC; Mayo endoscopic subscore of 2 + modified Mayo score of 4-6 + rectal bleeding subscore ≥1) and in the overall population who were advanced therapy (AT)–naive or exposed only to 5-aminosalicylic acid (5-ASA) and who entered the OLE as clinical nonresponders at True North week 10 (observed case [OC] analysis). (A) Symptomatic response. (B) Symptomatic remission.

More week 10 nonresponders who were AT-naive with moderate UC compared with those in the overall AT-naive population achieved a delayed response after 5 weeks (69.0% and 53.5%, respectively) of additional ozanimod treatment; response after 10 weeks of treatment was similar between the 2 groups (62.1% and 62.4%, respectively) (Figure 6A). The differences between AT-naive patients with moderate UC and overall AT-naive patients were further pronounced when evaluating symptomatic remission (Figure 6B). Patients exposed only to 5-ASA were more likely to achieve symptomatic remission than AT-naive patients with moderate UC (Figure 6B). No differences were observed in sensitivity analyses with alternative definitions of moderate UC (OC analysis) (Supplemental Figure 7). Similar trends in symptomatic efficacy were observed in the NRI analyses (Supplemental Figure 8).

### Safety

The overall incidences of treatment-emergent adverse events (TEAEs) during the OLE were similar between AT-naive patients with moderate UC and patients exposed only to 5-ASA, with fewer serious TEAEs in those exposed only to 5-ASA (13.7% vs 17.9%) (Supplemental Table 4). A sudden death occurred in an AT-naive patient with moderate UC who was treated with ozanimod, but this was determined to be unrelated to study drug. The most common TEAEs were COVID-19 and lymphopenia. No new safety signals were noted from the first year of treatment in the overall AT-naive group.^24^

## Discussion

This post hoc analysis of the phase 3 True North study and True North OLE study demonstrated that ozanimod was safe, effective, and durable in patients with moderate UC (defined as MES of 2, MMS of 4-6, and RBS ≥1) who were uncontrolled on conventional therapies (eg, 5-ASA, immunomodulators, corticosteroids). These results were seen whether ozanimod was initiated immediately after nonresponse to 5-ASA only or after use of immunomodulators and/or corticosteroids. Onset of action and early efficacy rates at the end of induction were markedly higher in patients with moderate UC than the overall AT-naive population, which included patients with severe UC. However, these differences were much less apparent over the long term. Sensitivity analyses using 2 alternative definitions of moderate UC yielded similar results, indicating that the inclusion of clinical symptoms in the definition of UC severity did not affect the results.

An analysis in AT-experienced patients with moderate UC was not conducted because our primary objective was to evaluate ozanimod efficacy in AT-naive patients who have failed conventional therapies but had not yet progressed to severe UC. Evaluating ozanimod in this analyzed population was clinically relevant because American Gastroenterological Association guideline updates in 2024 emphasize the importance of initiating higher-efficacy ATs, such as ozanimod, earlier in the treatment paradigm in patients who are AT-naive.^26^ Additionally, the American College of Gastroenterology 2025 guidelines recommend initiating S1P receptor modulators for induction of remission in patients with moderate to severe UC.^27^ The proportions of patients with moderate UC receiving ozanimod were higher in the AT-naive population (∼49%) than the AT-experienced population (26%-29%) at True North baseline.^24^^,^^28^ A previous True North and True North OLE analysis in AT-experienced patients demonstrated that prior biologic exposure initially attenuates treatment response to ozanimod, further highlighting the importance of our analysis of AT-naive patients with moderate UC.^28^

In a practical review article, it was recommended that ozanimod be initiated before biologics, especially in patients with moderate UC.^29^ Our findings of enhanced ozanimod efficacy used early in the treatment paradigm, especially in patients with moderate UC immediately after failure of 5-ASA, are consistent with this positioning recommendation and are aligned with 2024 American Gastroenterological Association and 2025 American College of Gastroenterology guideline updates.^26^^,^^27^ Patients with moderate UC treated with ozanimod immediately after failure of 5-ASA demonstrated a rapid onset of symptomatic response after 2 weeks of treatment initiation. There were slightly higher rates of symptomatic response and symptomatic remission when ozanimod was administered directly after failure of 5-ASA than after nonresponse to 5-ASA and immunomodulators and/or corticosteroids. Patients who initiated ozanimod immediately after failure of 5-ASA were more likely to achieve all clinical and endoscopic efficacy endpoints compared with placebo at week 10, and these responses were maintained at week 52. Importantly, efficacy outcomes were sustained for approximately 5 years of continuous ozanimod treatment after failure of 5-ASA. For patients who did not achieve clinical response after 10 weeks of ozanimod therapy, extended induction treatment with ozanimod was shown to be beneficial. Specifically, a large proportion of patients who were delayed responders to ozanimod achieved symptomatic response and symptomatic remission with an additional 5 to 10 weeks of therapy. Even in the patients who were week 10 nonresponders, patients with moderate UC had an advantage over patients with moderate to severe UC. Our results align with those of a previous analysis showing that ∼50% to 60% and ∼18% to 24% of AT-naive nonresponders after 10 weeks of ozanimod treatment achieved symptomatic response and symptomatic remission, respectively, after an additional 5 to 10 weeks of ozanimod exposure.^24^

In a previous analysis of the overall AT-naive population with moderate to severe UC, ozanimod was well tolerated and had similar safety outcomes than those observed in the overall True North population.^24^ Notably, AEs of special interest (eg, bradycardia, malignancies, herpes zoster, serious infections) were low with ozanimod treatment in AT-naive patients with moderate to severe UC.^24^ Our safety results demonstrating extended continuous ozanimod treatment up to OLE week 190 were similar to those observed in a previous analysis of the True North OLE in all patients receiving continuous ozanimod for approximately 3 years.^25^ Reflective of these findings, an AT safety review article ranked S1P receptor modulators as one of the safest ATs for the treatment of inflammatory bowel disease.^30^ Our analysis further supports these results as ozanimod was well tolerated over approximately 5 years of continuous ozanimod treatment when initiated after 5-ASA.

Although many ATs are currently available for the treatment of UC, route of administration and tolerability are important factors that affect treatment choice.^12^^,^^31^^,^^32^ To many patients, biologics may not be ideal because they are infused or injected, and some have an unattractive safety profile from a patient’s perspective.^31^ Although Janus kinase inhibitors have an oral route of administration, they must be used after failure of ≥1 anti-tumor necrosis factor agent in the United States and may be associated with serious side effects, including increased risk of major adverse cardiovascular events, venous thromboembolism, and malignancy.^33^ Ozanimod has a favorable safety profile with a low incidence of these serious AEs.^23^ However, direct head-to-head trials between Janus kinase inhibitors and ozanimod are needed to fully compare safety profiles.

A limitation of our analysis is that True North was not designed or powered to explore only patients with moderate UC. Also, there was no control group allowing a comparison within the True North OLE. Results of this analysis may not be generalizable to the overall real-world AT-naive population of patients with moderate UC due to the stringent inclusion and exclusion criteria of clinical trials.

Despite these limitations, our analysis includes long-term data comprising approximately 5 years of ozanimod treatment from a large dataset and evaluates ozanimod efficacy using different definitions of moderate UC, an important and undertreated patient population in clinical practice. Unlike many prior randomized controlled OLE trials in IBD, the availability of endoscopic data that are included in the True North OLE provides insights into long-term, centrally read, objective endpoints, such as endoscopic improvement, histologic remission, and mucosal healing.

## Conclusions

This analysis demonstrated that ozanimod was effective, durable, and safe for up to ∼5 years in AT-naive patients with moderate UC who had active disease while on conventional therapy. This included the patients who started ozanimod immediately after failure of 5-ASA without initiating immunomodulators or corticosteroids. These results support the use of ozanimod as a first-line AT in patients with moderate UC in whom 5-ASA therapy fails.

## Supplementary Material

izaf195_Supplementary_Data

## Data Availability

Bristol Myers Squibb policy on data sharing may be found at https://www.bms.com/researchers-and-partners/independent-research/data-sharing-request-process.html. De-identified individual patient data will not be shared.
