# Supplementary material for: Long-Term Ozanimod Therapy in Patients With Moderately Active Ulcerative Colitis After Failure of 5-Aminosalicylic Acid
Source: Inflamm Bowel Dis. 2025 Sep 26;32(1):77–86. doi: 10.1093/ibd/izaf195 (PMC12759051; doi:10.1093/ibd/izaf195)
Supplement: izaf195_Supplementary_Data [file izaf195_supplementary_data.docx]

**SUPPLEMENTARY DATA**

### **Supplementary Methods**

### Study Design

True North was a randomized, double-blind, placebo-controlled, 52-week, pivotal phase 3 study that evaluated the efficacy and safety of ozanimod 0.92 mg in patients with moderate to severe ulcerative colitis (UC).^1^ True North consisted of a 10-week induction period (IP) and a 42-week maintenance period (MP). In the 10-week IP, patients were randomized 2:1 to receive double-blind, once-daily ozanimod 0.92 mg or placebo in Cohort 1; other patients received open-label ozanimod 0.92 mg in Cohort 2. Patients who received ozanimod underwent a 1-week dose escalation (ozanimod 0.23 mg on days 1–4, ozanimod 0.46 mg on days 5–7, and ozanimod 0.92 mg thereafter) before starting the ozanimod 0.92 mg daily dose. Patients receiving ozanimod who achieved clinical response at week 10 were rerandomized 1:1 to receive double-blind ozanimod 0.92 mg or placebo until the end of the 42-week MP. Patients who did not achieve clinical response at week 10, experienced disease relapse (ie, partial Mayo score ≥4 points or a ≥2-point increase from week 10 and an endoscopy subscore of ≥2 points) in the MP, or completed week 52 were eligible to enter the open-label extension (OLE).^1,2^ Patients who entered the OLE from a double-blind treatment group (ie, Cohort 1 in the IP and all MP groups) underwent the 1-week dose escalation upon initiation of ozanimod 0.92 mg. Patients who entered the OLE from open-label Cohort 2 continued to receive once-daily ozanimod 0.92 mg.

Patients

Patients aged 18–75 years with moderate to severe UC (ie, total Mayo score of 6–12 with a Mayo endoscopy subscore ≥2, rectal bleeding subscore ≥1, and a stool frequency subscore ≥1) were included. Patients were required to be receiving stable doses of oral 5-aminosalicylic acid, corticosteroid (prednisone ≤20 mg/day or budesonide), or both for ≥2 weeks before screening and throughout induction at the same dose; corticosteroid doses were tapered prior to entering maintenance. Patients discontinued immunomodulators (ie, azathioprine, 6-mercaptopurine, or methotrexate) prior to randomization, and concurrent treatment with immunomodulators was prohibited after randomization.

Statistical analysis

### In the True North IP, statistical comparisons of efficacy endpoints were performed in Cohort 1 and descriptive statistics were performed in Cohort 2; in the MP, statistical comparisons of efficacy endpoints were performed to compare the ozanimod treatment group with the group that switched from ozanimod to placebo. Treatment differences, 2-sided 95% Wald confidence intervals, and P-values were reported for comparison between the ozanimod and placebo groups for efficacy outcomes assessed at True North weeks 10 and 52 using the Cochran-Mantel-Haenszel test at a 5% significance level, stratified by corticosteroid use at screening for the induction analyses, and by week 10 clinical remission status and week 10 corticosteroid use for the maintenance analyses. Nominal P-values were reported for analyses that were not prespecified and multiplicity-controlled in True North. Patients with missing efficacy data in the True North IP and MP were considered nonresponders using nonresponder imputation analysis.

### OLE data were summarized descriptively using observed case (OC) and nonresponder imputation (NRI) analyses, and no hypothesis testing was performed. OC analyses included patients with data available for the endpoints in question at each timepoint, and missing data were excluded. NRI analyses included patients who completed each timepoint or discontinued ozanimod therapy, and patients with missing data were considered nonresponders.

######

## Supplementary Table 1. Endpoint definitions

| **Efficacy endpoints** | **Definition** |
| --- | --- |
| Symptomatic response | Decrease from baseline in the combined 6-point RBS + SFS of ≥1 point and ≥30%, and decrease of ≥1 point in RBS or absolute RBS ≤1 |
| Symptomatic remission | RBS of 0 and SFS ≤1 (and a decrease of ≥1 point from the baseline SFS) |
| Clinical remission | RBS of 0, SFS ≤1 (and decrease of ≥1 point from baseline SFS), and MES ≤1 |
| Clinical response | Decrease from baseline in the modified Mayo score (sum of RBS, SFS, and MES) of ≥2 points and ≥35%, and decrease of ≥1 point in RBS or absolute RBS ≤1 |
| Endoscopic improvement | MES ≤1 without friability |
| Histologic remission | Geboes score <2.0 |
| Mucosal healing | MES ≤1 and histological remission |
| Corticosteroid-free remission | Clinical remission while off corticosteroid for ≥12 weeks |
| Endoscopic remission | MES = 0 |

Abbreviations: MES, Mayo endoscopy subscore; RBS, rectal bleeding subscore; SFS, stool frequency subscore.

**Supplementary Table 2.** Baseline demographic and clinical characteristics in the overall AT-naive population (moderate/severe UC) and in AT-naive patients with moderate UC using alternative definitions of moderate disease

| **Characteristics** | **Overall AT-naive patients (moderate/severe UC)^3^**  **(n=616)** | | | **AT-naive patients with moderate UC** | | | | | |
| --- | --- | --- | --- | --- | --- | --- | --- | --- | --- |
|  |  |  |  | **MES = 2** | | | **MES = 2 + TMS^a^ 6–9 + RBS ≥1** | | |
|  | **Cohort 1** | | **Cohort 2** | **Cohort 1** | | **Cohort 2** | **Cohort 1** | | **Cohort 2** |
|  | **Placebo (n=137)** | **Ozanimod**  **(n=287)** | **Ozanimod**  **(n=192)** | **Placebo**  **(n=64)** | **Ozanimod**  **(n=138)** | **Ozanimod**  **(n=94)** | **Placebo**  **(n=62)** | **Ozanimod**  **(n=127)** | **Ozanimod**  **(n=86)** |
| Age, y, mean (SD) | 42.7 (13.8) | 41.8 (13.3) | 44.0 (13.9) | 42.6 (13.6) | 41.9 (13.2) | 42.9 (14.9) | 42.7 (13.7) | 42.5 (13.2) | 43.2 (15.1) |
| Male, n (%) | 90 (65.7) | 164 (57.1) | 118 (61.5) | 38 (59.4) | 78 (56.5) | 54 (57.4) | 38 (61.3) | 74 (58.3) | 49 (57.0) |
| Years since UC diagnosis, mean (SD) | 6.1 (7.2) | 5.7 (5.9) | 7.0 (8.0) | 6.3 (7.0) | 5.5 (5.9) | 6.0 (7.4) | 6.2 (6.9) | 5.8 (6.1) | 5.9 (7.3) |
| Extent of UC disease, n (%)  Left-sided  Extensive | 90 (65.7)  47 (34.3) | 194 (67.6)  93 (32.4) | 139 (72.4)  53 (27.6) | 48 (75.0)  16 (25.0) | 97 (70.3)  41 (29.7) | 73 (77.7)  21 (22.3) | 46 (74.2)  16 (25.8) | 88 (69.3)  39 (30.7) | 69 (80.2)  17 (19.8) |
| TMS,^a^ mean (SD) | 8.7 (1.4) | 8.8 (1.4) | 8.8 (1.5) | 7.9 (1.1) | 7.9 (1.2) | 7.8 (1.2) | 7.8 (1.0) | 7.8 (1.0) | 7.7 (1.0) |
| MMS,^b^ mean (SD) | 6.5 (1.2) | 6.6 (1.2) | 6.6 (1.3) | 5.8 (1.0) | 5.9 (1.1) | 5.8 (1.1) | 5.7 (1.0) | 5.8 (0.9) | 5.7 (1.1) |
| MES, n (%)  Moderate (MES = 2)  Severe (MES = 3) | 64 (46.7)  73 (53.3) | 138 (48.1)  149 (51.9) | 94 (49.0)  98 (51.0) | 64 (100.0)  0 | 138 (100.0)  0 | 94 (100.0)  0 | 62 (100.0)  0 | 127 (100.0)  0 | 86 (100.0)  0 |
| Concomitant 5-ASA use, n (%) | 128 (93.4) | 271 (94.4) | 186 (96.9) | 60 (93.8) | 132 (95.7) | 93 (98.9) | 58 (93.5) | 123 (96.9) | 85 (98.8) |
| Concomitant CS use at baseline, n (%) | 39 (28.5) | 63 (22.0) | 44 (22.9) | 18 (28.1) | 23 (16.7) | 20 (21.3) | 16 (25.8) | 20 (15.7) | 16 (18.6) |
| Prior 5-ASA use, n (%) | 134 (97.8) | 281 (97.9) | 192 (100.0) | 62 (96.9) | 135 (97.8) | 94 (100.0) | 60 (96.8) | 124 (97.6) | 86 (100.0) |
| Prior CS use, n (%) | 89 (65.0) | 188 (65.5) | 123 (64.1) | 42 (65.6) | 92 (66.7) | 57 (60.6) | 40 (64.5) | 83 (65.4) | 52 (60.5) |
| Prior IMM use, n (%) | 33 (24.1) | 77 (26.8) | 34 (17.7) | 15 (23.4) | 27 (19.6) | 12 (12.8) | 15 (24.2) | 26 (20.5) | 12 (14.0) |

Abbreviations: 5-ASA, 5-aminosalicylic acid; AT, advanced therapy; CS, corticosteroid; IMM, immunomodulator; MES, Mayo endoscopy subscore; MMS, modified Mayo score; PGA, Physician Global Assessment; RBS, rectal bleeding subscore; SFS, stool frequency subscore; TMS, total Mayo score; UC, ulcerative colitis.

^a^Sum of RBS, SFS, MES, and PGA subscore.

^b^Sum of RBS, SFS, and MES.

**Supplementary Table 3.** Baseline demographic and clinical characteristics in the overall population of patients exposed only to 5-ASA (moderate/severe UC) and patients exposed only to 5-ASA with moderate UC using alternative definitions

| **Characteristics** | **Overall patients exposed only to 5-ASA (moderate/severe UC)**  **(n=375)** | | | **Patients exposed only to 5-ASA with moderate UC** | | | | | | | |
| --- | --- | --- | --- | --- | --- | --- | --- | --- | --- | --- | --- |
|  |  |  |  | **MES = 2** | | | | **MES = 2 + TMS^a^ 6–9 + RBS ≥1** | | | |
|  | **Cohort 1** | | **Cohort 2** | **Cohort 1** | | **Cohort 2** | **Cohort 1** | | | **Cohort 2** |  |
|  | **Placebo**  **(n=80)** | **Ozanimod**  **(n=170)** | **Ozanimod**  **(n=125)** | **Placebo**  **(n=37)** | **Ozanimod**  **(n=95)** | **Ozanimod**  **(n=65)** | **Placebo**  **(n=37)** | | **Ozanimod**  **(n=87)** | **Ozanimod**  **(n=61)** |  |
| Age, y, mean (SD) | 43.4 (13.7) | 42.3 (13.3) | 44.2 (13.9) | 43.3 (13.6) | 41.8 (12.8) | 42.4 (14.5) | 43.3 (13.6) | | 42.6 (12.9) | 42.1 (14.6) |  |
| Male, n (%) | 48 (60.0) | 94 (55.3) | 70 (56.0) | 20 (54.1) | 51 (53.7) | 32 (49.2) | 20 (54.1) | | 48 (55.2) | 30 (49.2) |  |
| Years since UC diagnosis, mean (SD) | 6.5 (8.1) | 4.9 (5.5) | 6.2 (7.7) | 7.1 (8.1) | 4.7 (5.6) | 6.1 (7.8) | 7.1 (8.1) | | 4.9 (5.7) | 6.0 (7.8) |  |
| Extent of UC disease, n (%)  Left-sided  Extensive | 56 (70.0)  24 (30.0) | 118 (69.4)  52 (30.6) | 98 (78.4)  27 (21.6) | 30 (81.1)  7 (18.9) | 68 (71.6)  27 (28.4) | 53 (81.5)  12 (18.5) | 30 (81.1)  7 (18.9) | | 62 (71.3)  25 (28.7) | 51 (83.6)  10 (16.4) |  |
| TMS,^a^ mean (SD) | 8.5 (1.3) | 8.6 (1.4) | 8.6 (1.4) | 7.7 (1.0) | 7.9 (1.2) | 7.8 (1.2) | 7.7 (1.0) | | 7.7 (1.0) | 7.7 (1.0) |  |
| MMS,^b^ mean (SD) | 6.4 (1.2) | 6.4 (1.2) | 6.4 (1.2) | 5.6 (1.0) | 5.8 (1.1) | 5.7 (1.1) | 5.6 (1.0) | | 5.7 (1.0) | 5.7 (1.0) |  |
| MES, n (%)  Moderate (MES = 2)  Severe (MES = 3) | 37 (46.3)  43 (53.8) | 95 (55.9)  75 (44.1) | 65 (52.0)  60 (48.0) | 37 (100.0)  0 | 95 (100.0)  0 | 65 (100.0)  0 | 37 (100.0)  0 | | 87 (100.0)  0 | 61 (100.0)  0 |  |
| Concomitant 5-ASA use, n (%) | 80 (100.0) | 169 (99.4) | 125 (100.0) | 37 (100.0) | 94 (98.9) | 65 (100.0) | 37 (100.0) | | 86 (98.9) | 61 (100.0) |  |
| Concomitant CS use at baseline, n (%) | 0 | 0 | 0 | 0 | 0 | 0 | 0 | | 0 | 0 |  |
| Prior 5-ASA use, n (%) | 79 (98.8) | 167 (98.2) | 125 (100.0) | 36 (97.3) | 92 (96.8) | 65 (100.0) | 36 (97.3) | | 84 (96.6) | 61 (100.0) |  |
| Prior CS use, n (%) | 34 (42.5) | 81 (47.6) | 57 (45.6) | 16 (43.2) | 54 (56.8) | 29 (44.6) | 16 (43.2) | | 48 (55.2) | 28 (45.9) |  |
| Prior IMM use, n (%) | 0 | 0 | 0 | 0 | 0 | 0 | 0 | | 0 | 0 |  |

Abbreviations: 5-ASA, 5-aminosalicylic acid; AT, advanced therapy; CS, corticosteroid; IMM, immunomodulator; MES, Mayo endoscopy subscore; MMS, modified Mayo score; PGA, Physician Global Assessment; RBS, rectal bleeding subscore; SFS, stool frequency subscore; TMS, total Mayo score; UC, ulcerative colitis.

^a^Sum of RBS, SFS, MES, and PGA subscore.

^b^Sum of RBS, SFS, and MES.

**Supplementary Table 4.** Safety summary in patients with moderate UC in True North Week 52 responders during the OLE

| **Preferred Term, n (%)** | **AT-naive patients with moderate UC**  **(n=67)** | **Patients exposed only to 5-ASA with moderate UC**  **(n=51)** |
| --- | --- | --- |
| TEAE | 52 (77.6) | 40 (78.4) |
| Related TEAE | 1 (1.5) | 1 (2.0) |
| Serious TEAE | 12 (17.9) | 7 (13.7) |
| TEAE leading to discontinuation | 3 (4.5) | 3 (5.9) |
| Deaths | 1 (1.5) | 0 |
| Most common TEAE^a^ |  |  |
| COVID-19 | 17 (25.4) | 11 (21.6) |
| Lymphopenia | 9 (13.4) | 8 (15.7) |
| Hypertension | 9 (13.4) | 4 (7.8) |
| Headache | 8 (11.9) | 6 (11.8) |
| Nasopharyngitis | 5 (7.5) | 5 (9.8) |
| Lymphocyte count decreased | 5 (7.5) | 0 |
| Hepatic enzyme increased | 4 (6.0) | 3 (5.9) |
| Arthralgia | 4 (6.0) | 2 (3.9) |

Abbreviations: AT, advanced therapy; CS, corticosteroid; IMM, immunomodulator; OLE, open-label extension; TEAE, treatment-emergent adverse event; UC, ulcerative colitis.

^a^Most common defined as ≥5% in patients in either group.

**Supplementary Figure 1.** True North and True North OLE study design. ^a^Patients were stratified by prior anti-TNF exposure and corticosteroid use (yes/no) at screening. ^b^Clinical response: a reduction from baseline of ≥1 point or absolute score of ≤1 point in RBS, plus a reduction of ≥2 points and ≥35% on the 3-component Mayo score, or ≥3 points and ≥30% on the 4-component Mayo score. ^c^Disease relapse: partial Mayo score increase ≥2 points vs the week 10 score and absolute score ≥4 points, endoscopy subscore of ≥2 points, and exclusion of other causes of an increase in disease activity unrelated to underlying ulcerative colitis. Abbreviations: OLE, open-label extension; RBS, rectal bleeding subscore; TNF, tumor necrosis factor. Reprinted with permission from Sandborn WJ et al. N Engl J Med. 2021;385:1280-1291.^1^


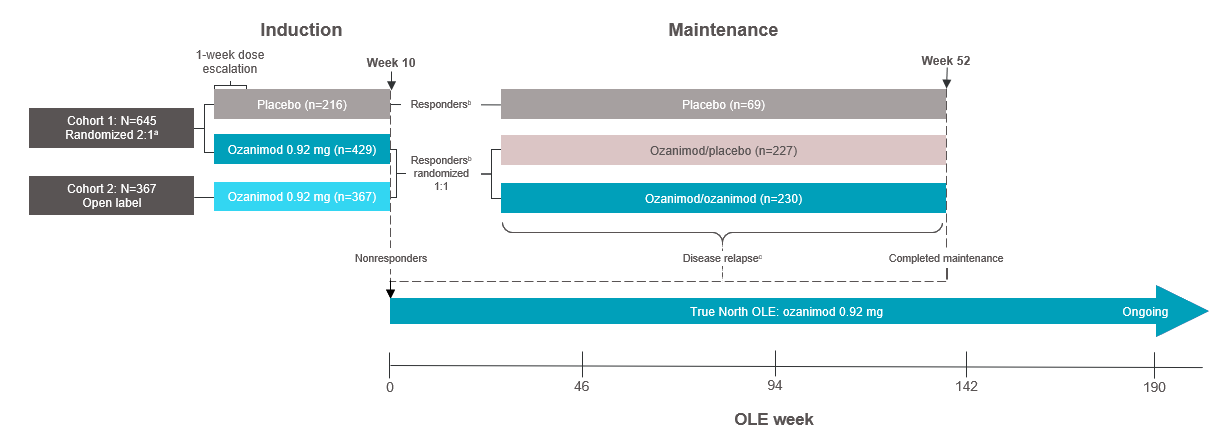


**Supplementary Figure 2.** Symptomatic response through week 10 in the induction period using alternative definitions of moderate UC: (A) MES = 2 and (B) MES = 2 + TMS = 6–9 + RBS ≥1. *P<0.05, **P<0.01, ***P<0.001 vs placebo. Shading indicates the 1-week dose-escalation period of ozanimod. Abbreviations: 5-ASA, 5-aminosalicylic acid; AT, advanced therapy; MES, Mayo endoscopy subscore; RBS, rectal bleeding subscore; TMS, total Mayo score; UC, ulcerative colitis.


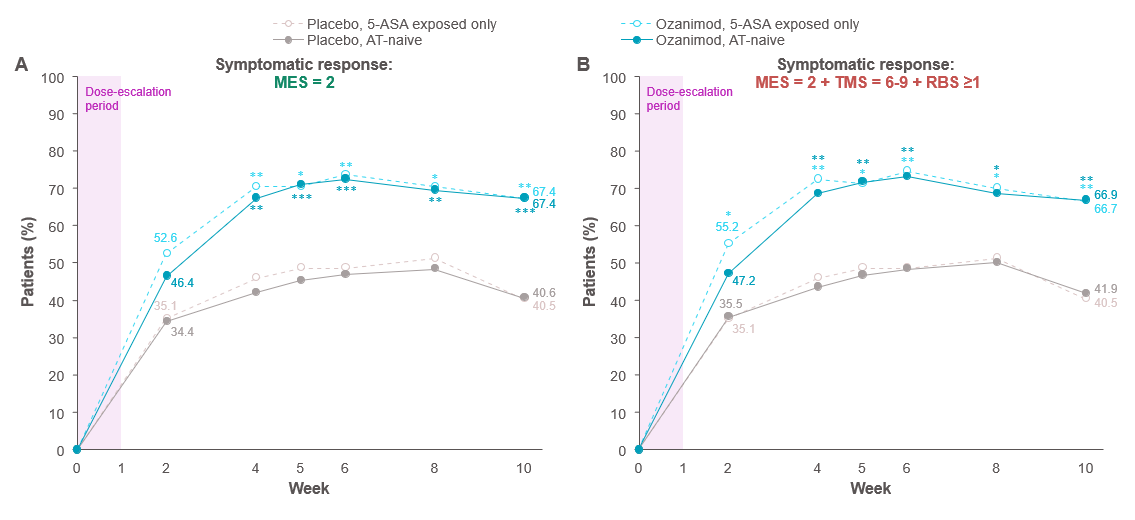


**Supplementary Figure 3.** Efficacy outcomes at week 10 using various definitions of moderate UC. (A) Clinical remission. (B) Clinical response. (C) Endoscopic improvement. (D) Mucosal healing. (E) Histologic remission. *P<0.05, **P<0.01, ***P<0.001 vs placebo. “∆” refers to the difference between ozanimod (Cohort 1) and placebo in the induction period. Abbreviations: 5-ASA, aminosalicylic acid; AT, advanced therapy; MES, Mayo endoscopy subscore; MMS, modified Mayo score; RBS, rectal bleeding subscore; TMS, total Mayo score; UC, ulcerative colitis.


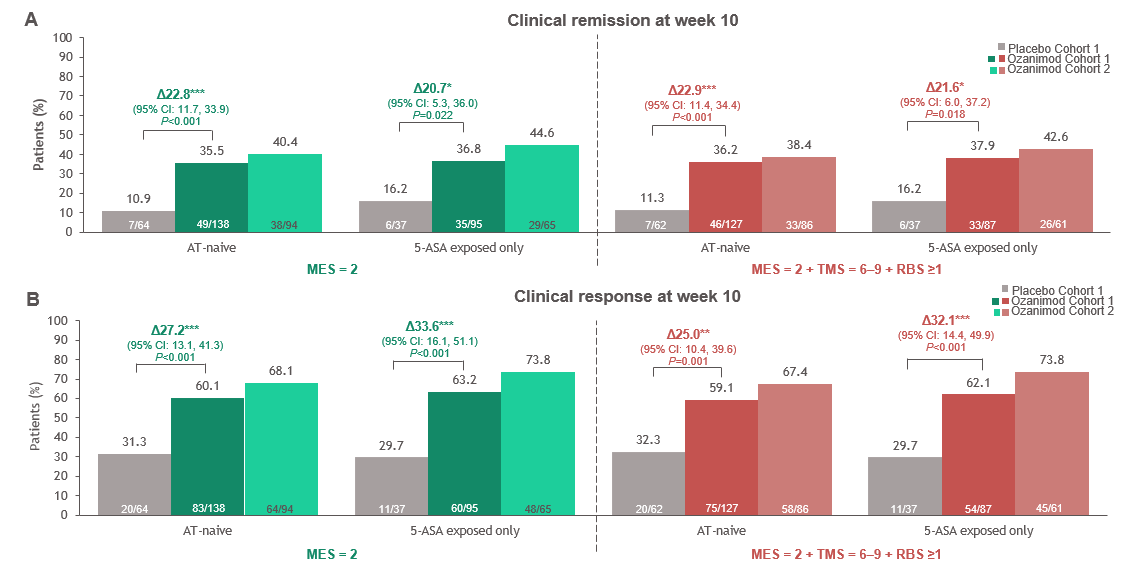

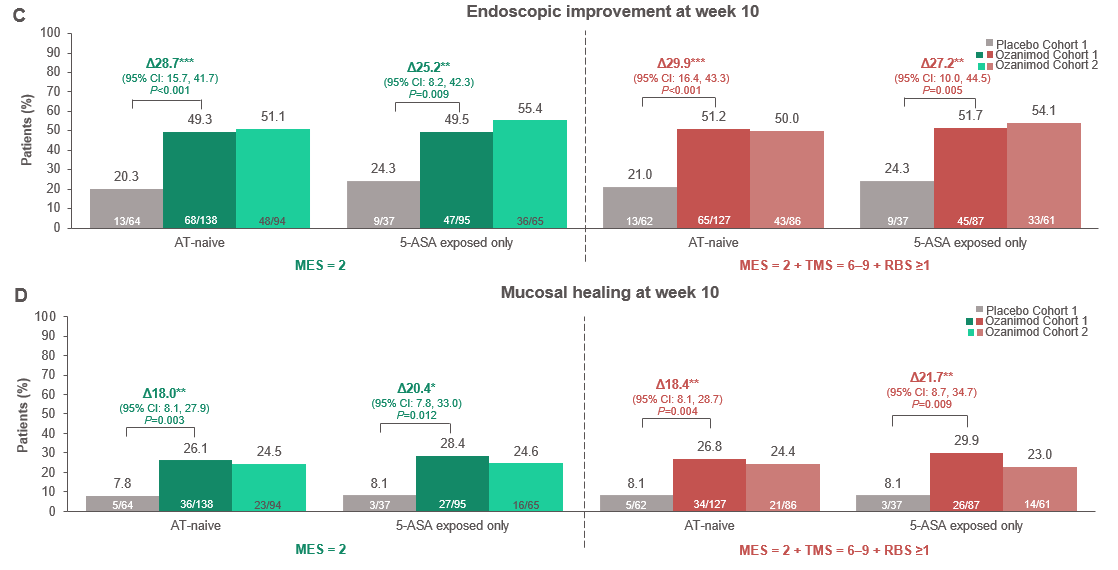


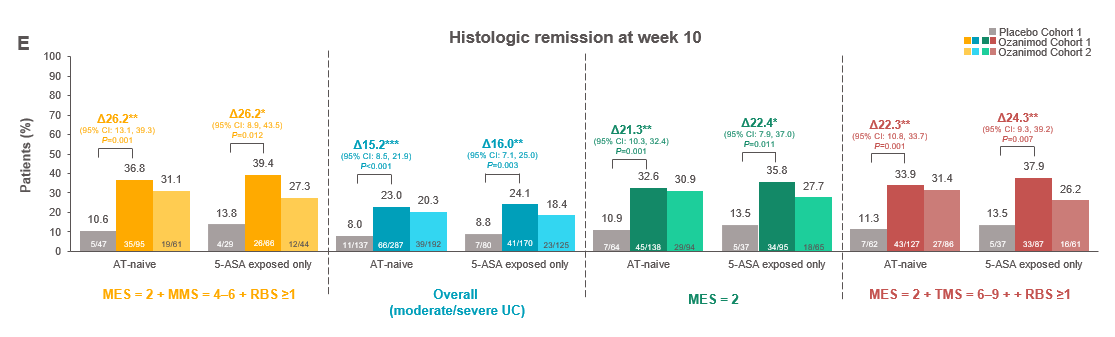


**Supplementary Figure 4.** Efficacy of outcomes at week 52 using various definitions of moderate UC. (A) CS-free remission. (B) Histologic remission. (C) Clinical remission. (D) Clinical response. (E) Endoscopic improvement. (F) Mucosal healing. *P<0.05, **P<0.01, ***P<0.001 vs placebo. Abbreviations: 5-ASA, 5-aminosalicylic acid; AT, advanced therapy; MES, Mayo endoscopy subscore; MMS, modified Mayo score; RBS, rectal bleeding subscore; TMS, total Mayo score; UC, ulcerative colitis.


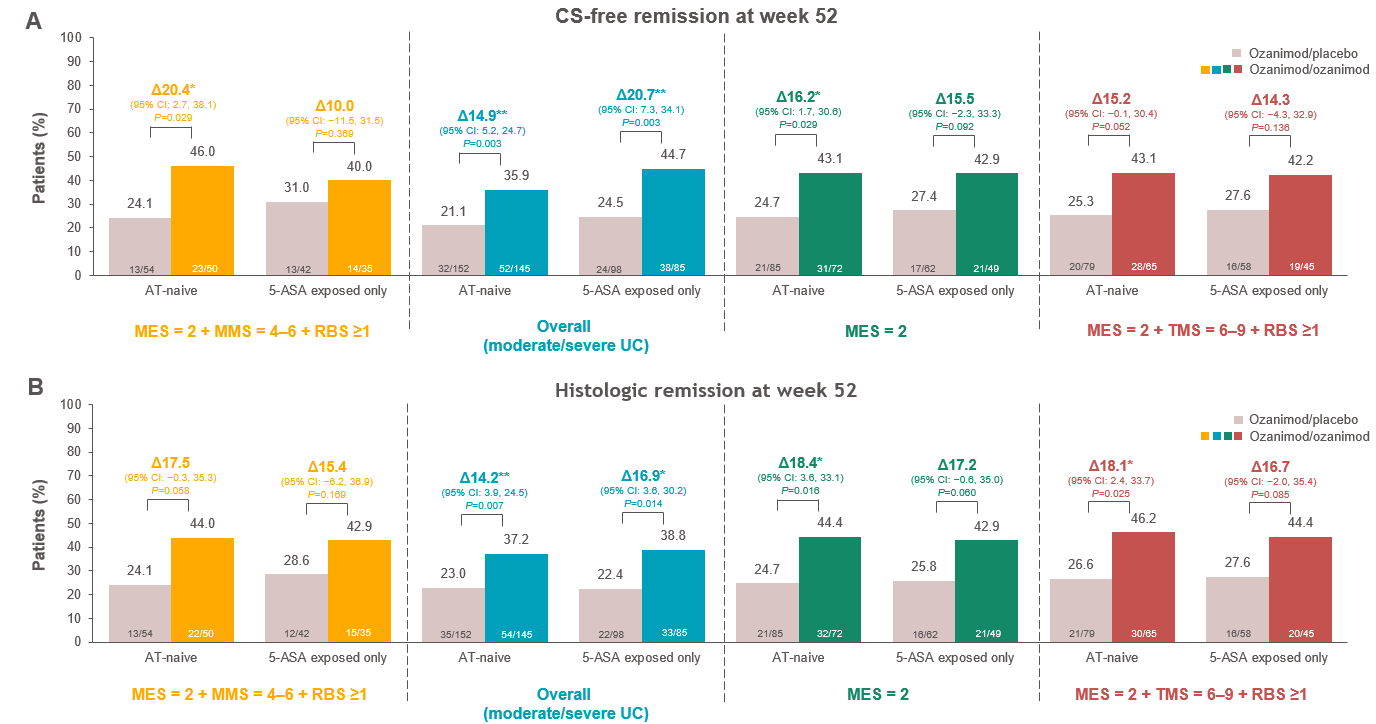


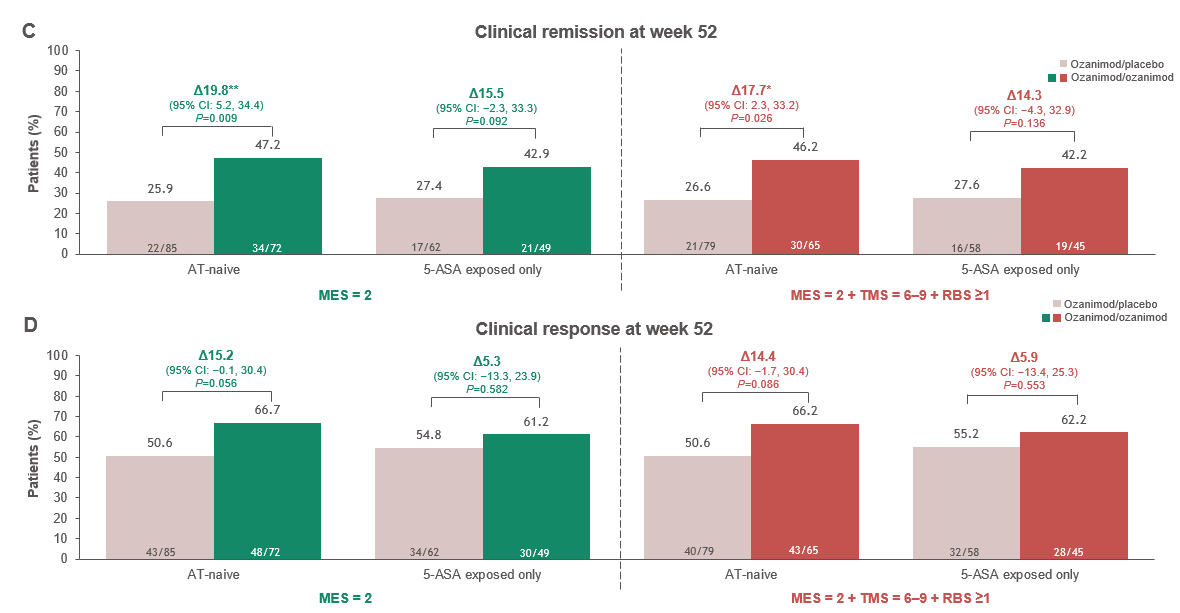


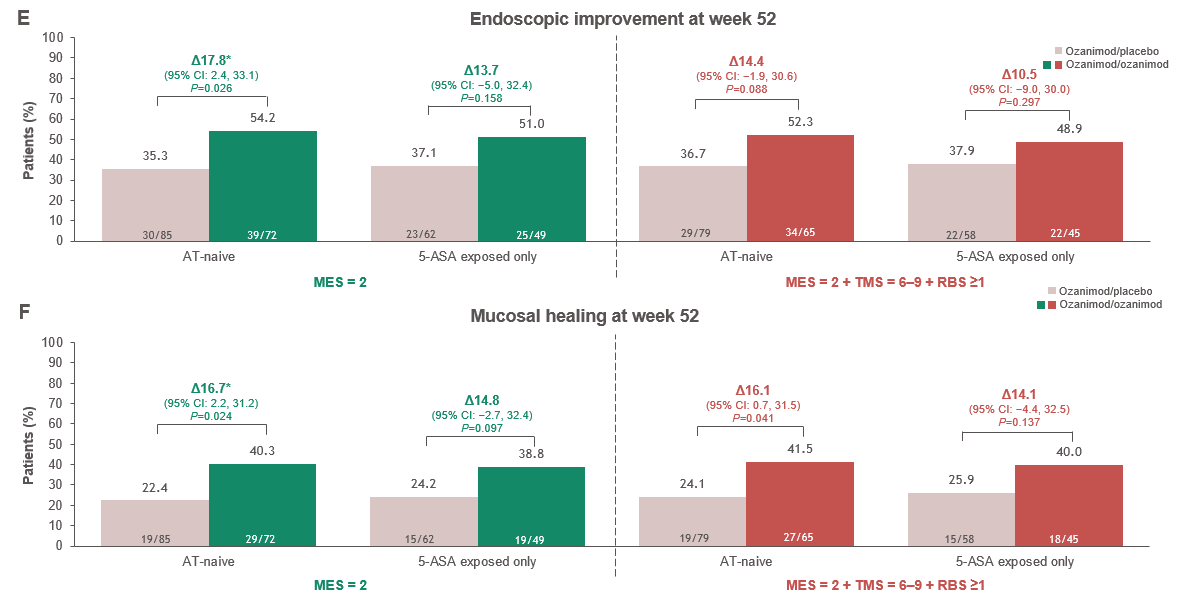


**Supplementary Figure 5.** Efficacy during the OLE in True North week 52 ozanimod clinical responders who continued on ozanimod in the OLE using various definitions of moderate UC (OC analysis). (A) Clinical remission. (B) Clinical response. (C) Endoscopic improvement. (D) Mucosal healing. (E) Histologic remission. (F) CS-free remission. (G) Endoscopic remission. Abbreviations: 5-ASA, 5-aminosalicylic acid; AT, advanced therapy; MES, Mayo endoscopy subscore; MMS, modified Mayo score; OC, observed case; OLE, open-label extension; RBS, rectal bleeding subscore; TMS, total Mayo score; UC, ulcerative colitis.


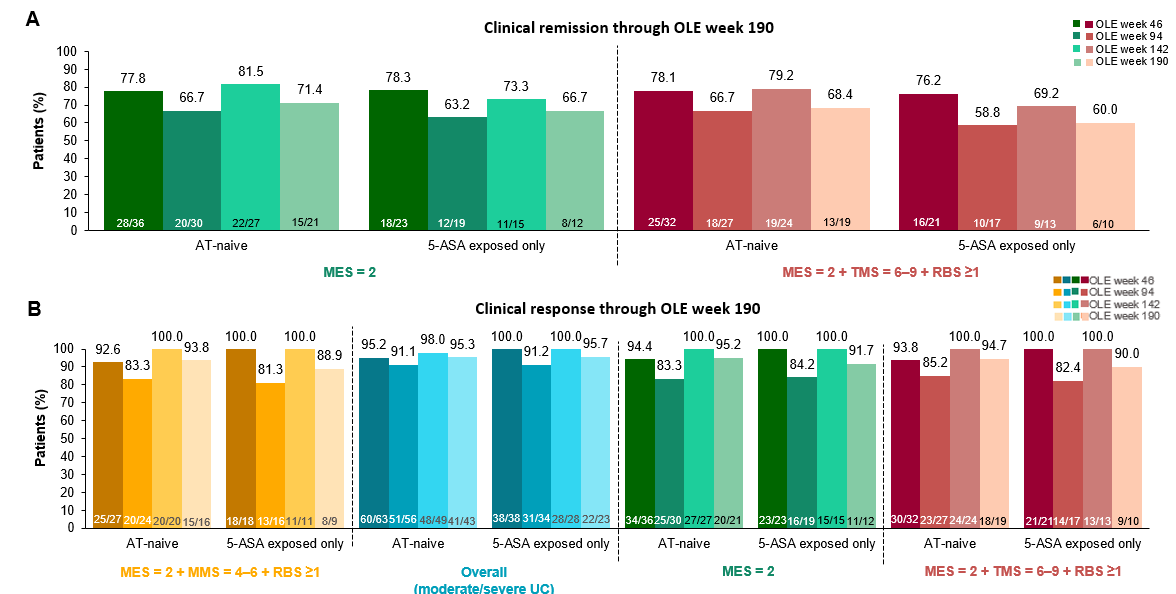


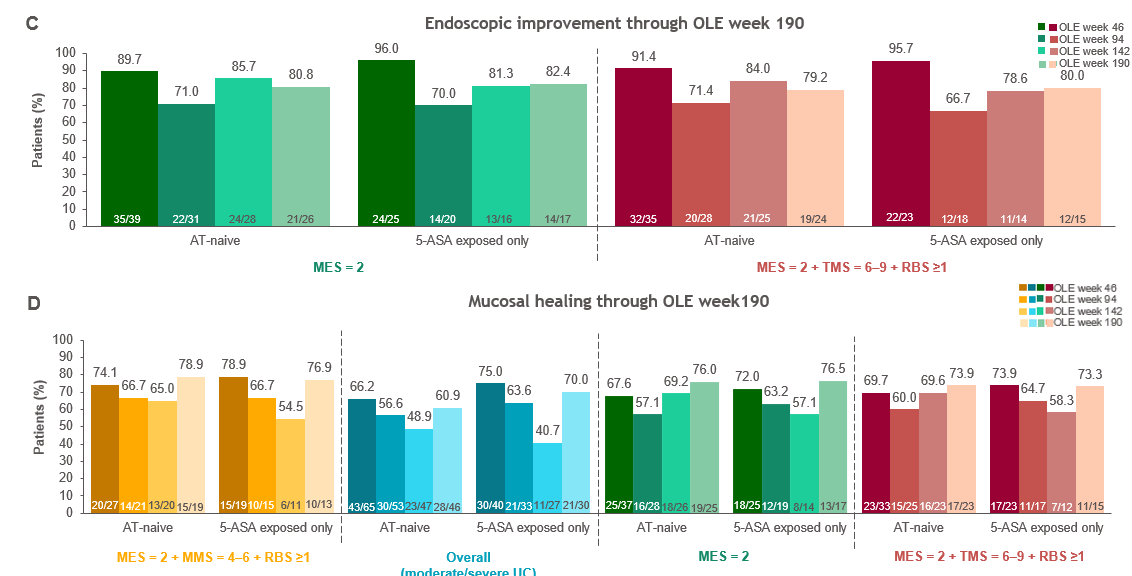

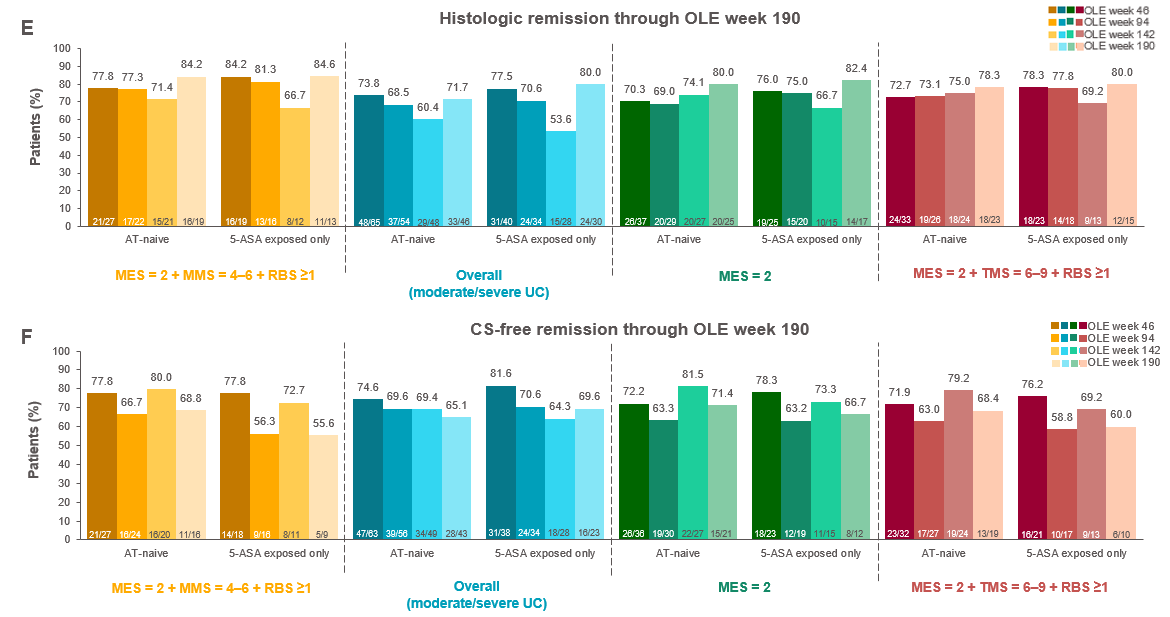


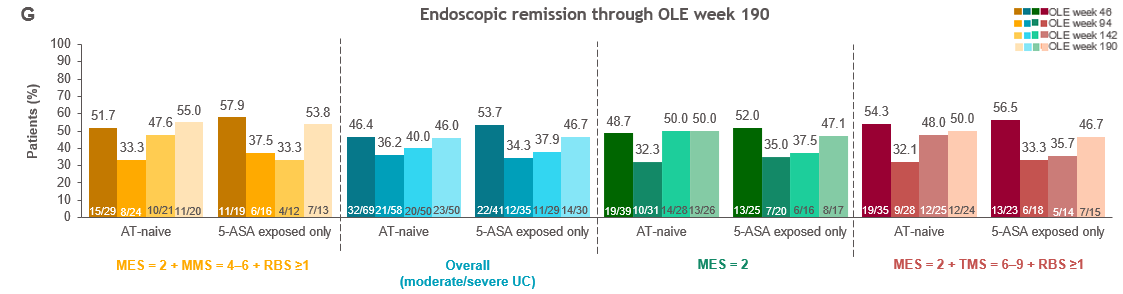


**Supplementary Figure 6.** Efficacy during the True North OLE using various definitions of moderate UC in patients who entered the OLE as True North week 52 ozanimod clinical responders (NRI analysis). (A) Clinical remission. (B) Clinical response. (C) Endoscopic improvement. (D) Mucosal healing. (E) Histologic remission. (F) CS-free remission. (G) Endoscopic remission. Abbreviations: 5-ASA, 5-aminosaliylic acid; AT, advanced therapy; MES, Mayo endoscopy subscore; MMS, modified Mayo score; NRI, nonresponder imputation; OLE, open-label extension; RBS, rectal bleeding subscore; TMS, total Mayo score; UC, ulcerative colitis
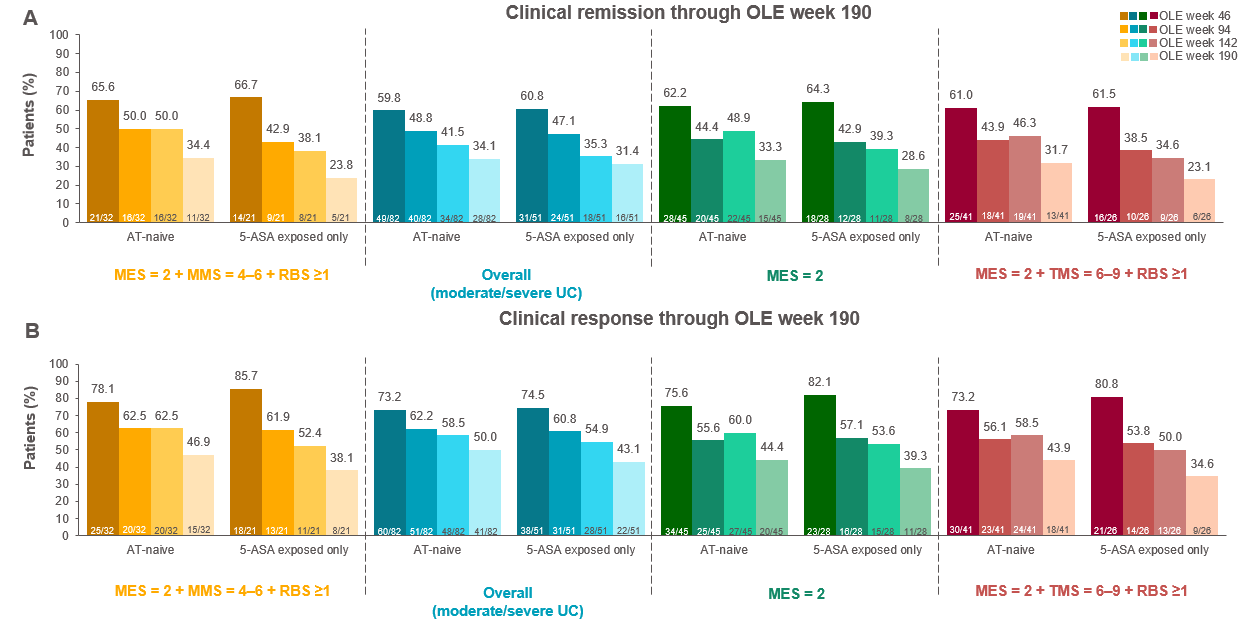

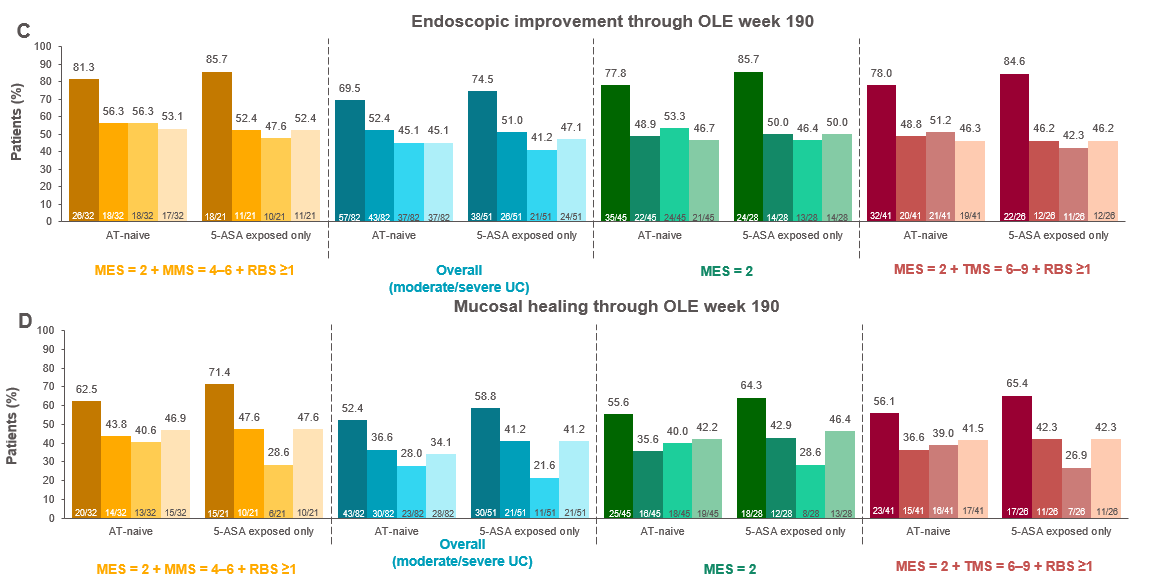


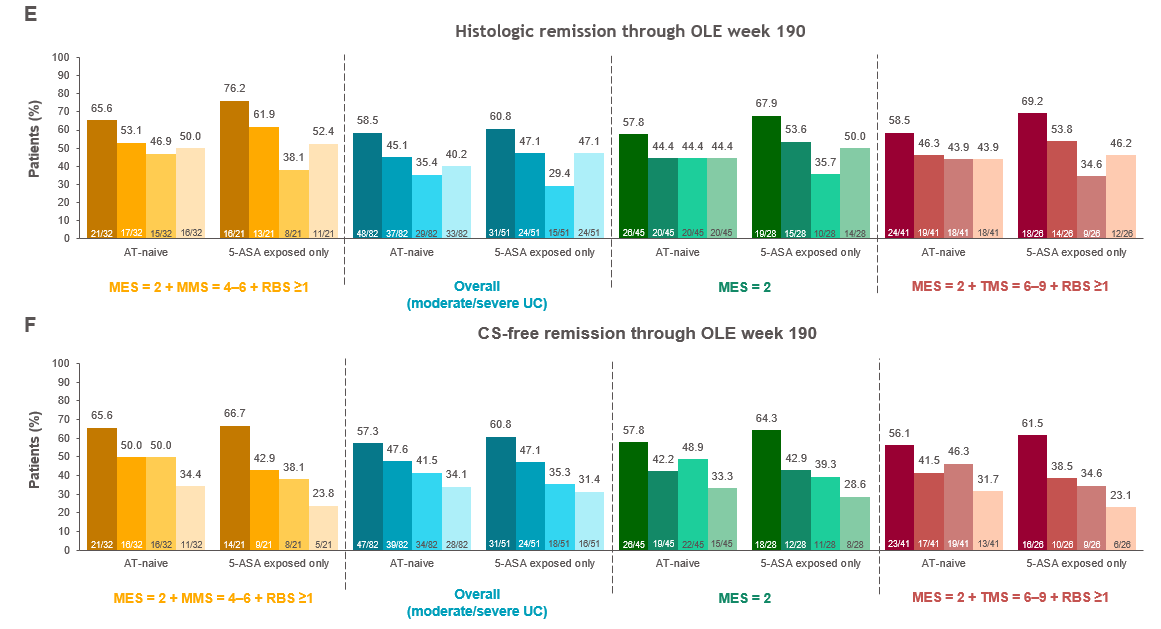


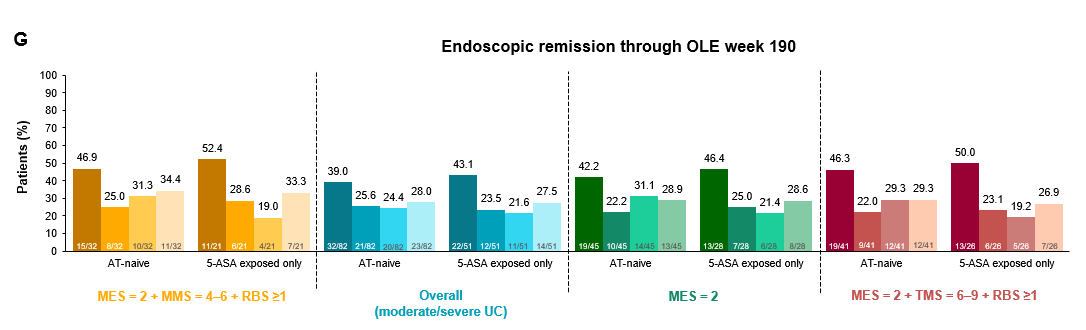


**Supplementary Figure 7.** Symptomatic efficacy during the OLE using various definitions of moderate UC in patients who entered the OLE as True North week 10 ozanimod clinical nonresponders (OC analysis). (A) Symptomatic response. (B) Symptomatic remission. Abbreviations: 5-ASA, 5-aminosalicylic acid; AT, advanced therapy; MES, Mayo endoscopy subscore; MMS, modified Mayo score; NRI, nonresponder imputation; OC, observed case; OLE, open-label extension; RBS, rectal bleeding subscore; TMS, total Mayo score; UC, ulcerative colitis.


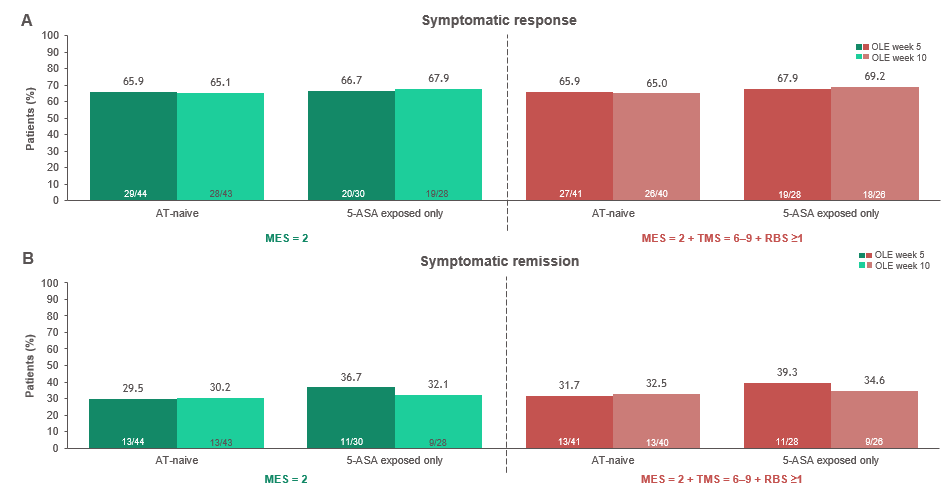


**Supplementary Figure 8.** Symptomatic efficacy during the OLE using various definitions of moderate UC in patients who entered the OLE as True North week 10 ozanimod clinical nonresponders (NRI analysis). (A) Symptomatic response. (B) Symptomatic remission. Abbreviations: 5-ASA, 5-aminosalicylic acid; AT, advanced therapy; MES, Mayo endoscopy subscore; MMS, modified Mayo score; NRI, nonresponder imputation; OC, observed case; OLE, open-label extension; RBS, rectal bleeding subscore; TMS, total Mayo score; UC, ulcerative colitis.


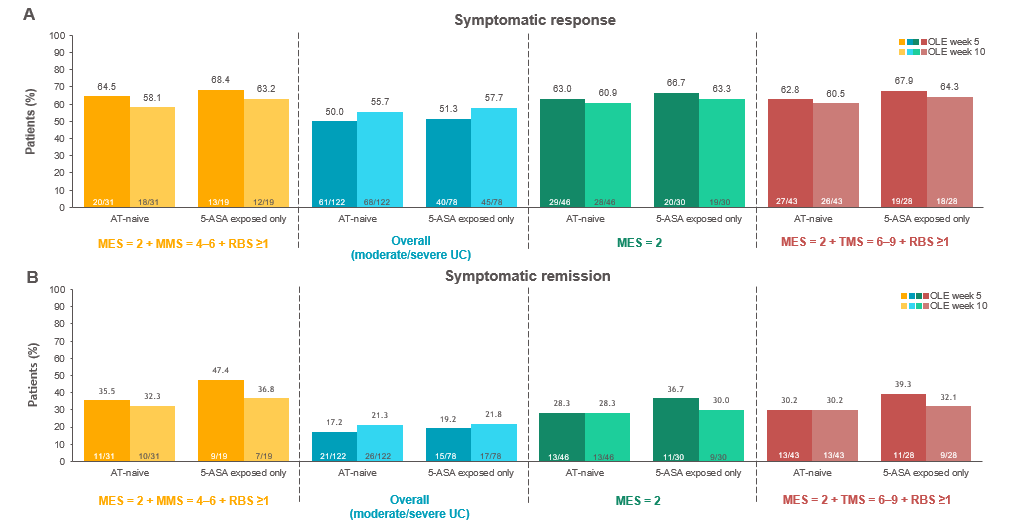


**References**

1. Sandborn WJ, Feagan BG, D'Haens G, et al. Ozanimod as induction and maintenance therapy for ulcerative colitis. N Engl J Med. 2021;385(14):1280-91.
2. Danese S, Panaccione R, Abreu MT, et al. Efficacy and safety of approximately 3 years of continuous ozanimod in moderately to severely active ulcerative colitis: interim analysis of the True North open-label extension. J Crohns Colitis. 2024;18(2):264-74
3. Sands BE, D'Haens G, Panaccione R, et al. Ozanimod in patients with moderate to severe ulcerative colitis naive to advanced therapies. Clin Gastroenterol Hepatol. 2024;22(10):2084-95.
